# Reasonable Cost for Procedures: An Anonymous Survey of Healthcare Providers

**DOI:** 10.36469/001c.143489

**Published:** 2025-09-08

**Authors:** David Shin, Carson Cummings, David Cheng, Chandler Dinh, Daniel Im, Timothy Tang, Isabella Oh, Lauren Han, Patricia Carlson, Gideon Harianja, Jacob Razzouk, Olumide Danisa, Wayne Cheng

**Affiliations:** 1 School of Medicine Loma Linda University, Loma Linda, California; 2 Socially Responsible Surgery Loma Linda University, Loma Linda, California; 3 School of Medicine Loma Linda University; 4 Orthopaedic Surgery Loma Linda University Medical Center, Loma Linda, California; 5 Orthopaedic and Neurological Surgery Duke University Health Care System, Durham, North Carolina; 6 Orthopaedic Surgery Jerry L. Pettis Memorial VA Medical Center, Loma Linda, California

**Keywords:** Medicare, Medicaid, lien, payment, economics, legal

## Abstract

**Background:**

The cost of medical procedures in the United States varies dramatically depending on the payment system, including Medicare, Medi-Cal (California’s Medicaid program), private insurance, or lien-based payment models used in personal injury cases. Cost discrepancies can discourage physician participation in Medicare and Medi-Cal, potentially limit access to care for vulnerable patient populations, and complicate the determination of proper compensation in court.

**Objectives:**

To survey healthcare providers to determine reasonable costs for medical procedures, potentially aligning legal standards with healthcare costs.

**Methods:**

An anonymous, 8-question electronic survey was distributed through Survey Legend® between February and September 2023 to providers in orthopedic surgery, neurosurgery, anesthesiology, interventional radiology (IR), physical medicine and rehabilitation (PMR), pain management, and physician assistants (PAs) or nurse practitioners (NPs). Three procedures—epidural injection, facet injection/medial branch block, and radiofrequency ablation—were included, with participants selecting from 5 cost categories: <1000,1000-4999,5000-9999,10000-19999,and>20,000. Additional questions explored participant insight into discounts for cash and lien-based payments.

**Results:**

For all procedures and participants, the most common value was 1000−4999. Neurosurgery selected significantly higher epidural values than pain management (*P*=.025), PMR (*P*=.029), and PA/NP (*P*=.04); higher facet injection/medial branch block values than PMR (*P*=.03) and PA/NPs (*P*=.01); and higher radiofrequency ablation values than PA/NPs (*P*=.02). Physicians not accepting lien payments showed significantly lower values across all specialties and procedures.

**Discussion:**

The range of reported reasonable costs by respondents reflects a discrepancy between physician expectations and existing reimbursement models, indicating a lack of a standardized value for procedural pricing. Medicare’s estimated 500reimbursementforepiduralinjectionsandfacetinjection/medialbranchblocksand1000 for radiofrequency ablation are below both physician-perceived reasonable costs and the inflated charges often found in lien-based cases. In contrast, personal injury billing can reach as high as $20,000 for an epidural injection, a cost category that only 2.9% of survey respondents chose.

**Conclusion:**

This survey highlights healthcare providers’ perceptions of reasonable costs for procedures, possibly assisting in refining reimbursement models, ensuring consistency in legal proceedings, and maintaining proper accessibility and compensation for patients and providers.

## INTRODUCTION

The cost of medical procedures in the United States varies dramatically depending on the payment system such as Medicare, Medi-Cal (California’s Medicaid program), private insurance, or lien-based payment models used in personal injury cases.^1^ A lien is defined as a legal right to a portion of an asset; typically, when physicians offer lien-based payments, the patient immediately receives treatment. The physician is then paid once the personal injury claim is settled or resolved.^2^ When Medicare or Medi-Cal covers the cost of medical care, the state can place a lien on any settlements or awards to recover expenses provided to the injured party. The California Department of Health Care Services typically asserts the lien through a claim and may pursue recovery through administrative or legal processes if necessary.^3^ In California, the Judicial Council of California Civil Jury Instruction 3903A establishes that plaintiffs in personal injury cases may recover the “reasonable cost of reasonably necessary medical care.”^4^ This process highlights the difficulties providers often face in reconciling the low reimbursement rates of Medicare or Medi-Cal with the significantly higher charges in lien-based personal injury cases.

For example, national averages of Medicare reimbursement for an epidural injection can be as low as $500, while costs in personal injury lien cases may reach up to $20 000 for the same procedure.^5–7^ Lien-based payments can reimburse physicians at a higher rate than their contractual rate with an insurer due to reasons including lack of pre-negotiated insurance rates, risk compensation, delayed payment, and legal negotiations.^2^ However, these discrepancies can discourage physician participation in Medicare and Medi-Cal and potentially limit access to care for vulnerable patient populations.^8^ Additionally, in cases where providers charge significantly higher rates, medical expenses may become inflated, increasing the burden on the medical and legal systems and complicating the determination of fair compensation in court. While defining a standard cost for medical care remains complex due to the wide range of reimbursement rates across different payment structures, such discrepancies underscore the importance of determining reasonable expenses for patient care, refining reimbursement models and compensation, and ensuring fairness and consistency in legal proceedings. To address these needs, this study aimed to explore what constitutes a reasonable cost for medical procedures through an anonymous survey of healthcare providers.

## METHODS

This study utilized an anonymous, self-administered electronic survey conducted through Survey Legend (SurveyLegend, 2025, Höör, Sweden). The survey was distributed via email to medical providers across multiple specialties, including orthopedic surgery, neurosurgery, anesthesiology, interventional radiology (IR), physical medicine and rehabilitation (PMR), pain management, and physician assistants (PAs) or nurse practitioners (NPs). These medical specialties were chosen due to their direct involvement in performing epidural injections, facet injections/medial branch blocks, and radiofrequency ablation. Survey participation was voluntary, and respondent anonymity was guaranteed by design. The survey was advertised through our institutions’ departmental mailing lists and was encouraged to be further spread via word of mouth. Data collection occurred between February and September 2023.

The 8-question survey aimed to assess healthcare providers’ perspectives on reasonable costs for various medical procedures, focusing on the influence of reimbursement models, specialty differences, and the impact of accepting lien-based payments. **Supplementary Table S1** details all questions provided by the survey. The first 2 questions gathered demographic information, including the respondent’s specialty and state of practice. The following 3 questions asked participants to estimate what they believed to be a reasonable cost for 3 standard procedures—epidural injection, facet injection/medial branch block, and radiofrequency ablation—considering all associated costs, such as the surgeon’s fee, facility fee, anesthesiologist fee, fluoroscopy, and supplies. For each of the 3 procedures, participants selected from 5 predefined cost categories: less than $1000, $1000-$4999, $5000-$9999, $10 000-$19 999, and more than $20 000. These categories were specified to evenly span the broad range of charges currently observed for these procedures, utilizing a distribution that captured low-end reimbursements like those from Medicare, and high-end charges seen in lien-based personal injury cases. The sixth question asked participants whether they would offer a discount for cash payments and, if so, what percentage they would be willing to provide. The 2 final questions regarding lien-based payments (1) assessed whether participants accepted lien arrangements and (2) examined the extent to which participants adjusted their charges under this payment model. After the closing date for questionnaire submissions, the results were downloaded as a CSV file for further analysis.

### Statistical Analysis

All data analyses were conducted using *R* version 4.4.3 (R Core Team, 2025, Vienna, Austria).^9^ Descriptive statistics were used to summarize the participant demographics, including specialty distribution (**Supplementary Table S2**). Due to an inadequate sample size (n = 2), IR specialty participants were excluded from analysis. The pain specialty group was also composed of both anesthesiologists and PMR, in addition to respondents who identified as only pain physicians, however no significant findings or differences were found when assessing for this component. Pairwise comparisons were conducted to assess the association between medical specialty and lien status vs reasonable value for: (1) epidural injections, (2) facet injections/medial branch blocks, and (3) radiofrequency ablation, as well as the reasonable discounts given to cash-paying patients. The Brant test was performed to test the parallel regression assumption, confirming that the assumption held for all analyses. Additionally, a model comparison was conducted to assess whether there was a significant interaction between specialty and lien status; the interaction was not statistically significant, so the main effects model was retained. Results were reported as odds ratios (ORs) with 95% confidence intervals (CIs), and statistical significance was set at *P* < .05.

## RESULTS

### Respondent Demographics

There were 98 survey responses recorded, with 30 responses excluded due to incomplete data, resulting in 68 surveys included in the final analysis. As displayed in **Supplementary Table S2**, respondents represented multiple medical specialties: 12 (17.6%) orthopedic surgery; 4 (5.9%) neurosurgery; 6 (8.8%) anesthesiology; 10 (14.7%) PMR; 28 (41.2%) pain management, which included anesthesiologists who had completed a pain fellowship; and 8 (11.8%) PAs or NPs. Among all specialties, 28 (41.18%) reported accepting lien-based payments, while 40 (58.82%) did not. Physicians practiced from a variety of locations: 57 (83.8%) in California, 7 (10.3%) in Hawaii, 3 (4.4%) in Minnesota, and 1 (1.5%) “other.”

### Estimated Reasonable Costs for Procedures

**Table 1** showcases the frequency of perceived reasonable values for each procedure. For all 3 procedures, the most common value was $1000-$4999, chosen by 37 (54.4%) for the reasonable cost of epidural injections, 37 (54.4%) for the reasonable cost of facet injection/medial branch blocks, and 42 (61.8%) for the reasonable cost of radiofrequency ablations. Notably, no respondents considered $10 000-$19 999 to be a reasonable cost for an epidural injection, and only 1 respondent (1.5%) selected this range for facet injections/medial branch blocks. For all 3 procedures, only 2 participants (2.9%) perceived costs to exceed $20 000.

**Table 1.** Frequency of Perceived Reasonable Values for Different Treatments

**Reasonable Values ($)**	**Epidural Injection, n (%)**	**Facet Injection/⁠Medial Branch Block, n (%)**	**Radiofrequency Ablation, n (%)**
<1000	20 (29.4)	19 (27.9)	4 (5.9)
1000-4999	37 (54.4)	37 (54.4)	42 (61.8)
5000-9999	9 (13.2)	9 (13.2)	17 (25.0)
10 000-19 999	0 (0)	1 (1.5)	3 (4.4)
>20 000	2 (2.9)	2 (2.9)	2 (2.9)

Neurosurgery was significantly associated with higher epidural values compared with pain (*P* = .025), PMR (*P* = .029), and PA/NP (*P* = .04) (**Table 2**). Neurosurgery was significantly associated with higher facet injection/medial branch block values compared with the specialties of PMR (*P* = .03) and PA/NPs (*P* = .01). (**Table 3**). Neurosurgery was significantly associated with higher radiofrequency ablation values compared with PA/NPs (*P* = .02) (**Table 4**). While pain physicians consisted of multiple specialties such as anesthesia and PMR who completed pain fellowships, subgroup analyses within pain yielded no significant findings.

**Table 2.** Pairwise Comparisons Using Ordinal Logistic Regression for Specialty Predicting Reasonable Epidural Injection Payment

**Reference Group**	**Predictor**	**OR**	**95% CI**		***P* Value**
**Lower Bound**	**Upper Bound**				
Pain	Anesthesiology	1.310	0.256	6.924	.746
	Neurosurgery	11.325	1.378	105.449	.025
	Orthopedic surgery	2.531	0.662	10.108	.179
	PMR	0.885	0.224	3.509	.861
	PA/NP	0.918	0.192	4.403	.913
Anesthesiology	Pain	0.764	0.144	3.911	.747
	Neurosurgery	8.655	0.761	108.773	.083
	Orthopedic surgery	1.934	0.309	12.295	.480
	PMR	0.677	0.104	4.291	.679
	PA/NP	0.702	0.093	5.139	.727
Neurosurgery	Anesthesiology	0.116	0.009	1.314	.083
	Pain	0.088	0.009	0.726	.025
	Orthopedic surgery	0.223	0.022	2.034	.183
	PMR	0.078	0.007	0.762	.029
	PA/NP	0.081	0.007	0.881	.040
Orthopedic surgery	Anesthesiology	0.517	0.081	3.235	.480
	Pain	0.395	0.099	1.511	.179
	Neurosurgery	4.474	0.492	44.993	.183
	PMR	0.350	0.068	1.731	.201
	PA/NP	0.363	0.060	2.109	.260
PMR	Anesthesiology	1.480	0.233	9.657	.677
	Pain	1.129	0.285	4.464	.861
	Neurosurgery	12.790	1.312	140.084	.029
	Orthopedic surgery	2.858	0.578	14.724	.201
	PA/NP	1.037	0.174	6.202	.968
PA/NP	Anesthesiology	1.427	0.195	10.706	.726
	Pain	1.090	0.227	5.207	.913
	Neurosurgery	12.339	1.135	149.945	.040
	Orthopedic surgery	2.758	0.474	16.653	.260
	PMR	0.965	0.161	5.753	.968
Lien	No lien	0.262	0.087	0.731	.013

Abbreviations: CI, confidence interval; NP, nurse practitioner; OR, odds ratio; PA, physician assistant; PMR, physical medicine and rehabilitation.

**Table 3.** Pairwise Comparisons Using Ordinal Logistic Regression for Specialty Predicting Reasonable Facet Injection/Medial Branch Block Payment

**Reference Group**	**Predictor**	**OR**	**95% CI**		***P* Value**
			**Lower Bound**	**Lower Bound**	
Pain	Anesthesiology	1.370	0.249	7.610	.717
	Neurosurgery	7.547	0.947	65.525	.056
	Orthopedic surgery	1.725	0.454	6.698	.424
	PMR	0.613	0.153	2.432	.485
	PA/NP	0.322	0.065	1.473	.149
Anesthesiology	Pain	0.730	0.131	4.023	.717
	Neurosurgery	5.508	0.485	67.901	.170
	Orthopedic surgery	1.259	0.194	8.327	.809
	PMR	0.447	0.064	3.020	.410
	PA/NP	0.235	0.029	1.769	.164
Neurosurgery	Anesthesiology	0.182	0.015	2.063	.170
	Pain	0.132	0.015	1.056	.056
	Orthopedic surgery	0.229	0.024	2.041	.184
	PMR	0.081	0.008	0.778	.030
	PA/NP	0.043	0.004	0.455	.010
Orthopedic surgery	Anesthesiology	0.794	0.120	5.146	.809
	Pain	0.580	0.149	2.201	.424
	Neurosurgery	4.374	0.490	41.990	.184
	PMR	0.355	0.069	1.750	.206
	PA/NP	0.187	0.030	1.053	.061
PMR	Anesthesiology	2.236	0.331	15.509	.410
	Pain	1.631	0.411	6.556	.485
	Neurosurgery	12.313	1.285	129.019	.030
	Orthopedic surgery	2.815	0.571	14.405	.206
	PA/NP	0.526	0.087	2.991	.471
PA/NP	Anesthesiology	4.253	0.565	34.418	.164
	Pain	3.103	0.679	15.458	.149
	Neurosurgery	23.424	2.196	278.485	.010
	Orthopedic surgery	5.356	0.950	33.052	.061
	PMR	1.902	0.334	11.519	.471
Lien	No lien	0.351	0.123	0.952	.044

Abbreviations: CI, confidence interval; NP, nurse practitioner; OR, odds ratio; PA, physician assistant; PMR, physical medicine and rehabilitation.

**Table 4.** Pairwise Comparisons Using Ordinal Logistic Regression for Specialty Predicting Reasonable Radiofrequency Ablation Payment

**Reference Group**	**Predictor**	**OR**	**95% CI**		***P* Value**
			**Lower Bound**	**Upper Bound**	
Pain	Anesthesiology	0.467	0.069	2.641	.408
	Neurosurgery	4.880	0.644	41.059	.126
	Orthopedic surgery	0.712	0.166	2.798	.634
	PMR	0.527	0.114	2.189	.391
	PA/NP	0.248	0.039	1.337	.120
Anesthesiology	Pain	2.142	0.379	14.436	.408
	Neurosurgery	10.452	0.891	146.492	.068
	Orthopedic surgery	1.525	0.209	12.348	.682
	PMR	1.129	0.147	9.354	.908
	PA/NP	0.532	0.055	5.113	.582
Neurosurgery	Anesthesiology	0.096	0.007	1.122	.068
	Pain	0.205	0.024	1.554	.126
	Orthopedic surgery	0.146	0.014	1.326	.091
	PMR	0.108	0.010	1.027	.057
	PA/NP	0.051	0.004	0.584	.020
Orthopedic surgery	Anesthesiology	0.656	0.081	4.795	.682
	Pain	1.405	0.357	6.021	.634
	Neurosurgery	6.855	0.754	70.728	.091
	PMR	0.740	0.128	4.176	.732
	PA/NP	0.349	0.046	2.426	.295
PMR	Anesthesiology	0.886	0.107	6.805	.908
	Pain	1.897	0.457	8.774	.391
	Neurosurgery	9.260	0.974	101.212	.057
	Orthopedic surgery	1.351	0.239	7.807	.732
	PA/NP	0.471	0.061	3.398	.460
PA/NP	Anesthesiology	1.881	0.196	18.199	.582
	Pain	4.028	0.748	25.455	.120
	Neurosurgery	19.658	1.712	266.405	.020
	Orthopedic surgery	2.868	0.412	21.876	.295
	PMR	2.123	0.294	16.425	.460
Lien	No lien	0.231	0.075	0.662	.008

Abbreviations: CI, confidence interval; NP, nurse practitioner; OR, odds ratio; PA, physician assistant; PMR, physical medicine and rehabilitation.

### Lien-Based Adjustments and Cash Discounts

Physicians with a “no-lien” status (ie, those who did not accept lien-based payments) were significantly associated with lower procedural values across all specialties and procedures. Specifically, those not accepting lien-based payments were approximately 73.8%, 64.9%, and 76.9% less likely to be in a higher payment category for epidural procedures, facet injections/medial branch blocks, and radiofrequency ablations, respectively (**Tables 2, 3, and 4**). Additionally, lien status had no significant association with cash discounts (**Table 4**). There was no significant association with a discount given to cash-paying patients across all specialties (**Table 5**). **Table 6** illustrates frequency of percent discount for cash payments for all procedures.

**Table 5.** Pairwise Comparisons Using Ordinal Logistic Regression for Specialty Predicting Reasonable Discount Given for Cash-Paying Patients

**Reference Group**	**Predictor**	**OR**	**95% CI**		***P* Value**
			**Lower Bound**	**Upper Bound**	
Pain	Anesthesiology	3.27	0.58	20.30	.18
	Neurosurgery	3.27	0.41	30.05	.26
	Orthopedic surgery	0.43	0.09	1.90	.27
	PMR	2.30	0.56	9.67	.25
	PA/NP	1.56	0.30	7.80	.59
Anesthesiology	Pain	0.31	0.05	1.72	.18
	Neurosurgery	1.00	0.08	12.84	1.00
	Orthopedic surgery	0.13	0.02	1.02	.06
	PMR	0.70	0.09	4.98	.72
	PA/NP	0.48	0.05	3.86	.49
Neurosurgery	Anesthesiology	1.00	0.08	12.14	1.00
	Pain	0.31	0.03	2.42	.26
	Orthopedic surgery	0.13	0.01	1.36	.09
	PMR	0.70	0.07	6.74	.76
	PA/NP	0.48	0.04	5.13	.54
Orthopedic surgery	Anesthesiology	7.52	0.98	64.48	.06
	Pain	2.30	0.53	10.57	.27
	Neurosurgery	7.52	0.74	89.51	.09
	PMR	5.29	0.90	33.42	.07
	PA/NP	3.58	0.52	25.81	.20
PMR	Anesthesiology	1.42	0.20	10.72	.72
	Pain	0.43	0.10	1.78	.25
	Neurosurgery	1.42	0.15	15.25	.76
	Orthopedic surgery	0.19	0.03	1.11	.07
	PA/NP	0.68	0.10	4.29	.68
PA/NP	Anesthesiology	2.10	0.26	18.63	.49
	Pain	0.64	0.13	3.30	.59
	Neurosurgery	2.10	0.19	25.70	.54
	Orthopedic surgery	0.28	0.04	1.94	.20
	PMR	1.48	0.23	9.80	.68
Lien	No lien	0.28	0.05	1.36	.12

Abbreviations: CI, confidence interval; NP, nurse practitioner; OR, odds ratio; PA, physician assistant; PMR, physical medicine and rehabilitation.

**Table 6.** Frequency of Percent Discount for Cash Payments

**Percent Discount**	**n (%)**
No discount	7 (10.3)
0-20	40 (58.8)
21-40	21 (30.9)

## DISCUSSION

This survey study examined physician perceptions of reasonable costs for epidural injections, facet injections/medial branch blocks, and radiofrequency ablation procedures across multiple specialties. Results were analyzed using pairwise comparisons to assess differences between specialties, lien-based payment status, and any trends with cash discounts. Our survey results suggest distinct variations in cost estimates based on both physician specialty and lien acceptance. Neurosurgeons reported higher reasonable values for all 3 procedures when compared with other certain specialties, such as pain management, PMR, and PA/NPs. Additionally, providers who did not accept lien-based payments consistently reported lower cost estimates across all 3 procedures. These findings complicate the legal determination of “reasonable” medical costs in personal injury cases, as California Civil Jury Instruction 3903A requires.^4^ Our results show significant variation in what constitutes a fair value for identical procedures within the medical community.^10^ This inconsistency poses challenges in evaluating the reasonableness of medical billing in litigation settings.

### Comparison With Existing Payment Structures

The range of reported reasonable costs by respondents highlights an ongoing discrepancy between physician expectations and existing reimbursement models. Medicare’s estimated $500 reimbursement for epidural injections and facet injection/medial branch blocks, as well as $1000 for radiofrequency ablation, represents one extreme of the pricing spectrum, as it is below both physician-perceived reasonable costs and the inflated charges often found in lien-based cases.^5,6,11,12^ In contrast, personal injury billing can reach as high as $20 000 for an epidural injection, a cost category that only 2.9% of survey respondents chose.^7^ The majority of responses were between these extremes, with responses most commonly (56.8%) falling into the $1000-$4999 range for all three procedures (**Table 1**). However, there were significant responses above and below this category. These findings reflect the broad variability in what providers perceive as reasonable and point to the lack of a standardized value for procedural pricing.^13^ The variability in perceived reasonable costs also raises concerns about how third-party payers, namely insurance companies, determine reimbursement rates that align with provider expectations. A more explicit definition of “reasonable cost,” in this context, would incorporate not just direct inputs like procedural supplies or physician pay, but also indirect costs such as administrative overhead or delayed payment risk and legal exposure in the case of lien-based payments.^2^ Expanded physician documentation could lead to more clarity as to what reasonable costs are and more accurate reimbursement.^14^ From the patient’s perspective, the absence of a consistent pricing framework under lien-based care may result in unpredictable medical costs and challenges in negotiating settlements. This ambiguity could result in exposure to inflated medical bills, reduced settlement proceeds, or protracted disputes over lien reductions.

### Impact of Specialty on Reasonable Cost Perception

Specialty-based differences were most prominent in comparisons involving neurosurgeons (**Tables 2, 3, and 4**), who consistently reported higher reasonable values for epidural injections, facet injections, and radiofrequency ablation procedures compared with pain management, PMR, and PA/NPs. The higher valuations from neurosurgeons may reflect a variety of factors. The length and rigor of neurosurgical training, with a potentially heightened perception of procedural and medicolegal risk, may elevate neurosurgeons’ internal idea of what constitutes fair compensation. Additionally, the fee-for-service relative-value-unit payment model may condition providers like neurosurgeons to view similar procedures through a higher-value lens, leading to the trends found in our results.^15^ Pairwise comparisons did not reveal statistically significant differences among most other specialties, suggesting that outside of neurosurgery, a relatively shared perception of procedural value exists among the other specialties administering pain management procedures.

### Effect of Lien and Cash Discounts on Cost Estimates

Across all 3 procedures, providers who reported not accepting lien-based payments were significantly less likely to report higher-value responses. Those not accepting liens were 73.8% less likely to report higher payment categories for epidurals, 64.9% less likely for facet injections, and 76.9% less likely for radiofrequency ablations. These trends suggest that physicians may adjust pricing based on anticipated legal and financial risk. Because compensation is contingent on the outcome of a personal injury claim, there is a substantial risk that providers may receive a reduced payment, or none at all, if the case is unsuccessful or settles for less than anticipated.^16^ Personal injury litigation is often prolonged, delaying reimbursement for months to years. To account for this delay and the associated opportunity cost, providers may preemptively raise their initial charges or inflate charges, ensuring that the final negotiated amount still meets their minimum financial expectations.^16^ All these factors play a part in the wide range and often inflated price of lien-based payments. Furthermore, these lien trends reflect a broader concern about how any non-traditional payment models can distort price transparency. Unlike Medicare or private insurance, lien payments are unpredictable, contingent upon case outcomes, and are frequently reduced through legal negotiation.^2^ Providers who do not accept liens may only utilize traditional payment pathways that do not hold payment uncertainty, explaining their more conservative perceptions.

The survey found no significant association between specialty or lien status and whether providers offered cash discounts. **Table 6** shows that 59% of physicians reported offering a 0% to 20% discount, 31% provided a 21% to 40% discount, and only 10% offered no discount. These findings suggest that cash discounting practices are idiosyncratic and likely influenced by local practice norms or patient demographics rather than systemic factors. This may indicate that cash discounting is driven more by individual or practice-level financial strategies than by broader forces like specialty norms or billing models. However, it remains imperative to refine medical reimbursement models to address discrepancies between insurance contracts and lien-based personal reimbursements. Such improvements may assist patients, particularly those who are underserved or economically disadvantaged, navigate payment strategies more effectively while also promoting fair treatment and equitable compensation.

### Limitations

This study has several limitations, primarily regarding the survey design and respondent demographics. The answer choices for reasonable costs were pre-determined categories, which may have limited the precision of responses. Free-text responses could have allowed participants to express more specific perceptions, resulting in more detailed estimates. Additionally, because the responses were self-reported perceptions rather than actual recorded costs, there is potential for bias, as physicians may have leaned toward ideal reimbursement values rather than actual current values.

Respondent demographics were also an important limiting factor. The included specialties did not cover all physicians who performed the surveyed procedures. Including additional specialties could broaden the range of perspectives, picking up on additional trends across different fields or practice settings. Some specialties were represented by far fewer respondents than others. For instance, only 4 neurosurgeons participated, in contrast to 28 pain management physicians. Specialties with smaller respondent numbers could have skewed our results and significant findings regarding neurosurgeons, as well as limited the ability to identify trends across specialties. Furthermore, the exclusion of 30 participants due to incomplete data is a significant number, and the lack of a formal power analysis to justify our sample size remains an important limitation regarding the generalizability of our findings.

Institutional differences and the geographic distribution of respondents may also have skewed responses. While the anonymity of the survey did not allow for the consideration of institutional differences, the majority of providers were located in California (83.8%). Healthcare systems, legal practices, costs of living, and cost expectations vary significantly by location and institution, so our results may not reflect national physician perspectives.^17^ A more geographically diverse sample would help broaden the study’s perspective and identify geographic trends in physician cost perceptions. Lastly, despite the widespread utilization of Medicare, this study did not consider any other public and private reimbursement plans. Future research should investigate additional comparisons between the vast and diverse selections of healthcare insurances to more accurately determine reasonable costs for procedures.

## CONCLUSION

This anonymous survey study examined physician and healthcare providers’ perceptions of reasonable costs for epidural injections, facet injections/medial branch blocks, and radiofrequency ablation procedures across multiple specialties. Results were analyzed using pairwise comparisons to assess differences between specialties, lien-based payment status, and any trends with cash discounts. For all 3 procedures, the most common perceived value for all participants was $1000 to $4999. Neurosurgery was associated with significantly higher epidural values than pain, PMR, and PA/NP; higher facet injection/medial branch block values compared with PMR and PA/NPs; and higher radiofrequency ablation values compared with PA/NPs. Physicians who did not accept lien-based payments showed significantly lower cost values across all specialties and procedures. Our findings offer insight into reasonable expenses for patient care, possibly assisting in refining reimbursement models and ensuring consistency in legal proceedings. Such improvements are imperative in assisting patients more effectively navigate payment strategies while also promoting fair treatment and equitable provider compensation. Future research may investigate comparisons between a broader range of public and private insurance contracts as well as lien-based personal reimbursements to better standardize cost estimates and allow for actionable and tangible policy adjustments.

## Supplementary Material

Online Supplementary Material
