# Supplementary material for: Reasonable Cost for Procedures: An Anonymous Survey of Healthcare Providers
Source: J Health Econ Outcomes Res. 2025 Sep 8;12(2):108–15. doi: 10.36469/001c.143489 (PMC12422406; doi:10.36469/001c.143489)
Supplement: Online Supplementary Material [file jheor_2025_12_2_143489_301288.pdf]

Journal of Health Economics and Outcomes Research

Online Supplementary Material

Reasonable Cost for Procedures: An Anonymous Survey of Healthcare Providers. *JHEOR*. 2025;12(2):108-115. doi:10.36469/jheor.2025.143489

Table S1: List of Survey Questions

Table S2: Participant Demographics

This supplementary material has been provided by the authors to give readers additional information about their work.

This is an open-access article distributed under the terms of the Creative Commons Attribution 4.0 International License (CCBY-4.0). View this license's legal deed at http://creativecommons.org/licenses/by/4.0 and legal code at http://creativecommons.org/licenses/by/4.0/legalcode for more information.

Table S1. List of Survey Questions

Question	Answer Choices
What is your specialty? Select all that apply.	Anesthesiology<br>Interventional radiology<br>Neurosurgery<br>Orthopedic surgery<br>Pain management<br>Physical medicine and rehabilitation<br>Physician assistant/nurse practitioner
In what state do you practice?	Hawaii<br>California<br>Minnesota<br>Other
What do you think is a reasonable dollar value for epidural injection (including surgeon's fee, facility fee, anesthesiologist fee, fluoroscopy, supplies, etc.)?	<$1000<br>$1000-$4999<br>$5000-$9999<br>$10 000-$19 999<br>>$20 000
What do you think is a reasonable dollar value for facet injection/medial branch block (including surgeon's fee, facility fee, anesthesiologist fee, fluoroscopy, supplies, etc.)?	<$1000<br>$1000-$4999<br>$5000-$9999<br>$10 000-$19 999<br>>$20 000
What do you think is a reasonable dollar value for radiofrequency ablation (including surgeon's fee, facility fee, anesthesiologist fee, fluoroscopy, supplies, etc.)?	<$1000<br>$1000-$4999<br>$5000-$9999<br>$10 000-$19 999<br>>$20 000
What percent (%) discount would you be willing to give if you are being paid in cash, if any?	No discount<br>0%-20%<br>21%-40%<br>40% and above
Do you accept payment on lien basis?	Yes<br>No
How much more would you charge if you are being paid on lien basis, if any?	No added charge<br>2 times the normal amount<br>3-5 times the normal amount<br>6-10 times the normal amount<br>>10 times the normal amount

Table S2. Participant Demographics

Variable	Category	n (%)
Specialty	Pain	28 (41.2)
	Anesthesiology	6 (8.8)
	Neurosurgery	4 (5.9)
	Orthopedic surgery	12 (17.6)
	Physician assistant/nurse practitioner	8 (11.8)
	Physical medicine and rehabilitation	10 (14.7)
Lien status	Yes lien	28 (41.2)
	No lien	40 (58.8)
State	California	57 (83.8)
	Hawaii	7 (10.3)
	Minnesota	3 (4.4)
	Other	1 (1.5)
**Total participants**		**68 (100)**
